# Obituary

**Published:** 1909-03-27

**Authors:** 


### OBITUARY.

The death occurred on March 16, at Staines, of Lieutenant-Colonel L. E. Fishbourne, R.A.M.C., M.D., F.R.C.S.I., at the age of 67

The death is announced to have taken place on March 16, at Eastbourne, after a few hours' illness, of Surgeon-Major Frederic Savignac Stedman, late of the Bombay Army, at the age of eighty.

Much sympathy is felt in the medical profession and outside it with Sir Richard Douglas Powell, the president of the Royal College of Physicians, upon his recent bereavement. Lady Powell, whose death took place on March 18 at Wimpole Street, was a daughter of the late Sir John Bennett, and was married to Sir R. Douglas Powell in 1873. One of her sons was killed in action in the Boer war.

The death is announced of Dr. James Hutchison Stirling, the well-known writer on philosophical subjects, whose 88th birthday prompted a short memoir in this column a year ago. He was born in 1820, and was educated at Glasgow University, and he spent six years in France and Germany. In early life he held appointments as surgeon to iron and coal works in South Wales, but in 1851 gave up practice and devoted himself to literature and philosophy. His publications included "The Secret of Hegel," "Sir William Hamilton, or the Philosophy of Perception," "Text-book to Kant," "Of Philosophy in the Poets," and "The Community of Property." In 1888 he was Gifford Lecturer to the University of Edinburgh, of which, as well as of Glasgow University, he was LL.D.

Dr. James Hurd Keeling, one of the leading medical men in Sheffield, consulting surgeon to the Sheffield Hospital for Women and to the Sheffield Royal Hospital, died on March 14. He was born in Malta in 1831, and studied medicine in Edinburgh, London, and abroad. He took the the F.R.C.S.Eng. diploma; and he served as a surgeon to the Turkish Army and in the Crimean war. He afterwards settled down in private practice near Sheffield. Moving into Sheffield in 1858, he was appointed lecturer on medical jurisprudence in the Sheffield Medical School in 1860, and lecturer on physiology in 1864. For forty years he was intimately connected with medical education in Sheffield, and it was largely through his efforts that the reorganisation of the Medical School in the year 1865 took place. He was one of the first medical officers connected with the Hospital for Women in Sheffield. He retired from practice in 1906.

The death took place early this month at Brentwood of Dr. John Cooper Quennell. Dr. Quennell, who was in his seventy-second year, held many public appointments, and was one of the oldest and best-known medical practitioners in Essex. He qualified in 1859, taking the diplomas of M.R.C.S.(Eng.) and L.S.A.(Lond.).

Captain Frederick Hallam Hardy, R.A.M.C., who died at Aden on March 8 of sleeping-sickness, on the voyage home from Nyasaland, was the son of Major-General F. Hardy, C.B., of Shawford, Winchester, and after studying at Guy's Hospital he went out to British Central Africa as a civil surgeon under the Foreign Office in 1898. He served in the Southern Angoniland expedition in 1898, and received the medal with clasp. In British Central Africa, in 1899, he took part in the expedition against Nkamba, and received the medal with clasp. He was gazetted to the Royal Army Medical Corps in June 1900, and received his commission in the King's African Rifles in 1901. In that year he took part in the expedition up the Gambia, and was mentioned in despatches and received the clasp. He was in East Africa in 1902-4 during the operations in Somaliland, and received two clasps. In 1906 Captain Hardy was seconded for service in British Central Africa under the Colonial Office, and whilst carrying out scientific research into the causes of tropical diseases he contracted sleeping-sickness, and the news of his serious illness was telegraphed home to his relatives early in January. He was placed in charge of Dr. Kinghorne, of the Liverpool School of Tropical Diseases, who was accompanying him to England. His untimely death recalls that of Lieutenant Forbes Tulloch, R.A.M.C., who died from the same cause, and under somewhat similar circumstances, only two or three years ago.

The Weather in 1908.—We have received from Mr. W. W. Wagstaffe, F.R.C.S., a carefully tabulated statement of the climatic and meteorological observations made by him at Sevenoaks during 1908. The figures given bear out the general impression existing among the public at large that during the past year the earlier months were both colder and rainier than usual; the summer finer on the whole, though the latter half of August was spoilt by cold and rain; and the late autumn quite unusually warm, fine, and dry. But it is evident that the variations from the normal, when expressed quantitatively, were a good deal less than would be expected: in fact that the actual difference between a good year and a bad one climatically is very slight. We note further that at Sevenoaks the total rainfall for the year was but .29 inch in excess of the average.

